# Write and Let Go: An Online Writing Program for University Students

**DOI:** 10.3389/fpsyg.2022.874600

**Published:** 2022-07-07

**Authors:** João Batista, Janine C. Marinai, Melissa Gouveia, João Tiago Oliveira, Miguel M. Gonçalves

**Affiliations:** Psychotherapy and Psychopathology Unit, School of Psychology, University of Minho, Braga, Portugal

**Keywords:** expressive writing, ambivalence, online intervention, writing based interventions, combined writing, positive writing, written disclosure paradigm

## Abstract

**Background:**

There are a plethora of studies on expressive writing and positive writing interventions, but few have addressed the combination of both paradigms. Additionally, research on the role of ambivalence toward change in the context of writing-based interventions is lacking. Ambivalence toward change is a natural movement of approaching and avoiding change that may occur in various situations. In psychotherapy, its resolution is associated with successful outcomes.

**Aim:**

This study tested the efficacy of a combination of expressive and positive writing paradigms in an internet-based intervention to improve university students’ mental health. Additionally, focusing participants on a current, unresolved problem allowed us to explore the possible role of ambivalence toward change as a mediator of the intervention’s results.

**Methods:**

We recruited 172 participants who were randomly divided into experimental (*n* = 85) and control (*n* = 87) groups. The intervention consisted of the identification of a current problem and four writing tasks on consecutive days. Assessment was conducted at baseline and posttest in both groups and at follow-up in the experimental group. Participants in the experimental condition were also assessed after each task. Measures of anxiety, depression, rumination, ambivalence toward change, distress, and wellbeing (optimism, affect, and satisfaction with life) were collected.

**Results:**

Multivariate analysis of variance (MANOVA) showed that participants in the experimental group had a significant decrease from baseline to posttest in ambivalence toward change and rumination when compared with the control group. These results were maintained at follow-up. No differences were found in the remaining measures. Within the experimental group, ambivalence toward change, rumination, and distress significantly decreased throughout the intervention and the exploratory mediation analysis indicated that ambivalence toward change partially mediated the improvements in rumination and distress.

**Discussion:**

Considering different perspectives about a current problem and using a combination of expressive and positive writing fostered the reduction of ambivalence toward change and rumination. Ambivalence toward change reduction after the second writing task may have created optimal conditions for the subsequent decrease in rumination and distress. Future studies should replicate this finding and dismantle the components that are more adequate in changing these variables.

## Introduction

This study introduces a writing-based program, termed *Write “n” Let Go*, using combined instructions to assist university students in reflecting on a current unresolved problem causing psychological distress. Departing from the considerable body of research on writing-based interventions (WBIs) (Wright and Chung, 2001), Write “n” Let Go articulated two major branches of research: expressive writing (EW) and positive writing (PW). Focusing on different facets of the problem was expected to help university students deal with their psychological distress, increase their wellbeing, and decrease symptoms, rumination, and ambivalence toward change. The program was implemented solely online, with the writing tasks performed autonomously, without face-to-face contact with the researchers.

To assess the efficacy of the program, we used measures of general wellbeing and symptoms, in addition to rumination as a cognitive factor and ambivalence towards change as a motivational factor. Previous studies with WBIs have shown improvements in rumination (Gortner et al., 2006; Glass et al., 2019), defined as a persistent and passive focus on the unrest and the meaning associated with depressive symptoms (Nolen-Hoeksema, 1998). Rumination is considered a thinking strategy to deal with difficulties common to several psychological disorders (Aldao et al., 2010) and a vulnerability factor for the appearance and maintenance of both depression (e.g., Teismann et al., 2014) and anxiety disorders (Watkins, 2004). Ambivalence toward change, on the other hand, is a novelty in the WBIs context. It can be defined as a conflict of opposite motivations, with one supporting change and another favoring maintenance of the *status quo*, coupled with distress (Urmanche et al., 2019; Oliveira et al., 2020, 2022). Previous WBIs studies have focused on other types of ambivalence (e.g., goal ambivalence, ambivalence over emotional expression) (Kelly et al., 2012; Heekerens et al., 2020).

Another feature of this study was the assessment of variables after each written task. We were particularly interested in examining the evolution of participants’ distress, rumination, and ambivalence toward change throughout the intervention. Additionally, since disclosure through writing can be considered a therapeutic process (Pennebaker, 1997), we explored whether ambivalence towards change could take part in such a process. By writing about a current problem, participants may be confronted with conflicting emotions regarding the need to change and the difficulty of doing so or the cost involved in it. Although dealing with ambivalence toward change *per se* does not guarantee a problem’s resolution, it may create motivation for a more adaptive way of dealing with it (Miller and Rollnick, 2002), potentially leading to reductions in rumination and distress associated with the problem, and improvements in symptoms and wellbeing.

### Paradigms of Writing-Based Interventions

Writing-based interventions have a long tradition in psychology, with low-cost interventions aimed at improving the physical and mental health of various populations. Interest in WBIs has been widespread, with at least 16 meta-analyses in this field, comprising hundreds of empirical studies. Although some meta-analyses failed to prove the efficacy of WBIs (Reinhold et al., 2018), several other meta-analyses (Smyth, 1998; Frattaroli, 2006) showed its efficacy in improving general psychological health. The disparity in the meta-analysis results can be explained by several factors, such as the number of studies included, the criteria for study selection (e.g., effects on specific populations), and the variables of interest (e.g., effects on either physical or psychological wellbeing). Moreover, studies in this field tend to use different ways of measuring the effects of interventions, from observational to self-report or psychophysiological measures.

The academic population has been a target of WBIs studies using varied formats. Pennebaker and Beall (1986) had students write about traumatic life events for 4 consecutive days, which they called the expressive writing (EW) paradigm, resulting in fewer health center visits in the 6 months following the experiment. Similarly, Robertson et al. (2019) managed to reduce depression using EW instructions applied to students transitioning to college with mild to severe symptoms. Alternatively, studies began exploring not only the difficulties generated by traumatic experiences but also the perceived benefits of those experiences (King and Miner, 2000). The positive writing (PW) paradigm further developed the notion that writing can be helpful to participants by focusing on intensely positive experiences (Burton and King, 2004), gratitude (Booker and Dunsmore, 2017), and strengths and competencies (Dolev-Amit et al., 2020), which showed a significant impact on participants’ wellbeing. In this paradigm, influenced by the positive psychology approach (Seligman, 2002), the awareness of the strengths and expression of positive emotions may lead participants to explore those aspects of themselves (Dolev-Amit et al., 2020). Similarly, the “best possible selves” (King, 2001) was developed as a way for participants to envision that their life goals would be achieved. In both procedures, it is supposed that attention to these positive aspects may have an impact on participants’ wellbeing.

Fewer experiments have tested the combination of EW and PW, which present participants with different instructions throughout the intervention. In one of these studies, King (2001) had a group of students write for 2 days about a traumatic life experience and the following 2 days about their best possible selves and did not obtain positive results from the intervention. Her conclusion was that with multiple instructions, the choice of topic is relevant and that it must be “meaningful, engaging, and even challenging. … one that can capture and maintain the individual’s attention for a considerable amount of time” (King, 2001, pp. 805/6). In another study, Lu and Stanton (2010) instructed students to focus on their current most stressful experience and divided the participants into four groups with different instructions, which were centered either on emotional disclosure, cognitive reappraisal, a combination of both, and a control group. A single relevant writing topic was the focus of all writing tasks. The combined instructions group was the most effective in reducing physical symptoms and increasing positive affect.

Despite the scarce research on the combination of paradigms, there is some evidence favoring the use of a combination of paradigms, vs. EW and PW individually (Lu and Stanton, 2010; Reinhold et al., 2018). Moreover, we considered it the best option for the Write “n” Let Go program because it allows participants to reflect on a topic of their choice from different perspectives, potentially eliciting various changing paths or processes. This notion of writing in a guided manner was also proposed by Gidron et al. (2002), with the Guided Disclosure Protocol that aimed to help participants explore different perspectives of a traumatic experience and accomplished successful results (Duncan and Gouchberg, 2007).

### Processes Involved in Writing-Based Intervention

Different processes have been proposed to explain how WBIs may operate to change participants’ health, mood, and wellbeing. It is considered that identifying the processes behind WBIs efficacy (or lack of it) could lead to interventions targeting core mechanisms associated with specific topics, populations, or psychological problems (Nazarian and Smyth, 2013). This proposal is similar to that of Kazdin (2009) regarding psychotherapy—understanding the processes involved in therapeutic change should allow optimization of interventions by identifying critical strategies and components.

EW effects for trauma-related topics have been mostly associated with two processes: *exposure* and *cognitive processing* (Frattaroli, 2006). Exposure is conceptualized in a similar way as in psychotherapy by a repeated description and confrontation with a negative experience and its emotional and cognitive consequences. Thus, writing about a traumatic experience may lead to habituation or the extinction of such feelings and thoughts (Sloan and Marx, 2004). Cognitive processing benefits are usually assessed through linguistic analysis and are associated with changes in traumatic event appraisal (King and Miner, 2000), an opportunity for participants to form a coherent story, make sense of the event, gain insights and integrate the upsetting experience into one’s self-schema (Pennebaker, 1993).

Another process, *self-regulation*, based on control theory, was proposed to explain the effects of both EW and PW (King, 2001). On EW, emotional disclosure may enhance emotional regulation through the mastery experience of observing ourselves and controlling our emotions while writing (Range and Jenkins, 2010). Writing may also lead to readjustments of goal-related emotions, which are drastically impacted by trauma, and increase self-regulation (Frattaroli, 2006). On PW, the raised awareness of values, strengths, and goals through self-observation can lead to increased self-efficacy and a sense of control over life’s challenges (Frattaroli, 2006). The “best possible self” interventions can foster a sense of self-efficacy for emotional regulation through mental simulation of successful outcomes (Frattaroli, 2006).

Despite the proposed processes, there is the assumption that none of them fully explains how writing works (Sloan and Marx, 2004). As Smyth and Pennebaker (2008) remarked, interacting factors likely drive its efficacy by impacting participants at different levels (physiological, emotional, cognitive, behavioral, and interpersonal). This assumption also corroborates our choice to use combined instructions, to foster different processes, promote their interaction, and increase the chances of a better fit to the participants’ needs. Considering the relevance of identifying the psychological processes underlying the WBIs’ effects and the use of a combined writing paradigm, our study also proposes to explore whether a motivational factor, here operationalized as ambivalence toward change, can contribute to this integrative context and help explain the effects of writing in cognitions and emotions.

### Ambivalence Toward Change

In almost every process of change, along with the desire and behaviors toward change we frequently present attitudes and/or behaviors in the opposite direction. This phenomenon has been conceptualized as ambivalence toward change and may occur in a variety of life situations. It involves a back-and-forth movement between opposite positions of the self, as an approach-avoidance conflict (Miller and Rollnick, 2002; Engle and Arkowitz, 2006; Gonçalves et al., 2011; Oliveira et al., 2016). Moreover, when the pathway toward change is challenged, maladaptive responses, such as rumination, may lead to distress and ultimately worsen symptomatology (Gilbert, 2001).

Several empirical studies have suggested that when ambivalence toward change emerges in the therapeutic change process, the person may often feel “of two hearts” about changing, which could lead to a feeling of being stuck (Oliveira et al., 2022). When the person is stuck regarding the process of change, the motivation to change tends to decrease (Emmons et al., 1993; Oliveira et al., 2022), increasing the subjective distress (Oliveira et al., 2020) and the probability of unsuccessful change (Miller and Rollnick, 2002; Oliveira et al., 2021b). In contrast, research demonstrates that individuals who change more consistently show a faster resolution of their ambivalence (Oliveira et al., 2021a). A major goal of therapeutic approaches targeting ambivalence toward change (Greenberg and Webster, 1982; Bents, 2006; Engle and Arkowitz, 2006; Miller and Rollnick, 2013) is to promote ambivalence resolution by raising awareness of the problem and the conflict between opposite motivations that lead to distress and the sense of being stuck.

As ambivalence toward change, to our knowledge, has not been investigated in the context of WBIs, we relied on studies that explored other forms of ambivalence. For instance, Kelly et al. (2012) proposed that goal ambivalence could be a target of WBIs. Their rationale was that if the resolution or acceptance of ambivalence in psychotherapy is due to awareness and insight, then writing about ambivalence should also reduce goal ambivalence and the associated distress. Kelly et al. (2012) used EW instructions and focused on an ambivalent goal, defined as a goal that can make the person unhappy even if the goal is achieved successfully. The intervention was successful in reducing distress about ambivalence. The study of Heekerens et al. (2020) departed from a similar argument, using the best possible self-exercise with instructions to write about the ideal future. The goal was to help students commit to some life goals at the expense of others, and the intervention resulted in a reduction in goal ambivalence and an increase in positive affect. These preliminary findings seem to indicate that goal ambivalence can be a relevant factor in WBIs.

Our choice, although, was to study ambivalence toward change (henceforth ambivalence) in a more naturalistic way, by asking participants to reflect on a current problem. Reflecting upon such problems and making adaptive changes imply some degree of awareness and motivation to handle difficult internal conflicts and the *pro*s and *cons* involved. Our expectation was that the writing tasks could lead to an increased awareness of both sides of ambivalence, which is, as referred, a target in ambivalence-centered interventions (Greenberg and Webster, 1982; Bents, 2006; Miller and Rollnick, 2013). By allowing the participants to view their problems from different perspectives, i.e., alternate the focus through the instructions provided, the intervention may prompt them to consider the conditions that maintain the problems and the adaptive changes that could lead to its acceptance or resolution. From this perspective, the combination of EW and PW instructions was the preferred option to promote participants’ reflections on their own, rather than using a single instruction. An increased awareness of the conflicting sides, from different perspectives, may increase their motivation to face the conflicts and harmonize the opposite sides involved in their problems, hence resolving ambivalence. Consequently, this could contribute to reducing rumination and distress associated with their difficulties and improving symptoms and wellbeing.

### Present Study

WBIs are inexpensive, easy to implement, and can reach a high number of participants. Thus, they are of great value in decreasing the risk of psychological suffering and increasing wellbeing. Although extensively studied, new formats such as online settings and the combination of paradigms are still under researched.

We developed an internet WBI combining EW and PW, named Write “n” Let Go. The program consisted of four 20-min writing tasks performed on consecutive days. The study design was based on the findings of a WBI meta-analysis (Reinhold et al., 2018), suggesting that larger effects were obtained with more intense interventions (more than three sessions) targeting a specific topic and using different instructions in each session. We opted for using four tasks, as having a greater number (six or eight) would be more demanding for participants, increasing the risk of experimental mortality. The choice for the 20-min duration was because the intervention was online and touched on current problems, so our goal was to mitigate the risk of overwhelming participants (Baikie and Wilhelm, 2005), while attempting to maximize the time to elaborate on the problem. Therefore, before writing, participants were asked to identify and describe a current problem in their lives that was causing psychological distress. This ensured that the writing was focused on a specific topic throughout the tasks and meant to make the experience as personal and meaningful as possible.

The combined instructions intended to promote different perspectives on the problem, leading to a better understanding of it, while seeking to balance problem activation (EW) and resource activation (PW), which in combination are considered a factor of progress in the context of psychotherapy (Gassmann and Grawe, 2006). In the negative emotion tasks (1 and 2), the mechanisms used for problem activation sought to foster exposure to difficult emotions, raise awareness of emotions, gain insights, make sense of or reappraise the problem, and form a coherent story (Pennebaker, 1993; Range and Jenkins, 2010). In the positive emotions tasks (3 and 4), resource activation sought to elicit positive emotions, raise awareness of values, strengths, and competencies, and simulate successful outcomes (Frattaroli, 2006). Gellaitry et al. (2010) tested a similar design applied to cancer patients, with positive results on satisfaction with emotional support. Their study also used 20-min writing tasks on 4 consecutive days and combined instructions that were based on the Guided Disclosure Protocol from King and Miner (2000) and Gidron et al. (2002).

The main objective of this study was to test the efficacy of a combined writing paradigm, assessing measures not only at pre/posttest, but also throughout the writing tasks. We tested three hypotheses:

Hypothesis 1: The writing program will reduce anxiety and depression symptoms, rumination, and ambivalence among participants in the experimental group compared with the control group, and this effect will be maintained at the follow-up.
Hypothesis 2: The writing program will increase the measures of wellbeing (satisfaction with life, optimism, and affective valence) in the participants of the experimental group compared with the control group, and this effect will be maintained at the follow-up.
Hypothesis 3: Ambivalence, rumination, and psychological distress will decrease throughout writing tasks.

An exploratory objective was to analyze the role of ambivalence toward change as a motivational process in the context of a WBI. More specifically, we were interested in testing whether a decrease in ambivalence at a given moment could mediate the WBI effects on symptoms, rumination, distress, or wellbeing.

## Method

### Participants

Participants were recruited between September 2020 and June 2021, after approval by the Research Ethics Committee in Social and Human Sciences at the University of Minho (process CEICSH 072/2020). The program was open to university students who were fluent in Portuguese and over 18 years old. The exclusion criteria were (a) presenting severe anxiety, measured with the Generalized Anxiety Disorder scale (cutoff score ≥ 15) (GAD-7; Spitzer et al., 2006), (b) presenting severe depression, measured with the Patient Health Questionnaire (cutoff score ≥ 20) (PHQ-9; Kroenke and Spitzer, 2002), and (c) presenting risk behaviors and/or suicidal ideation. These exclusion criteria were based on the notion that writing tasks may cause temporary discomfort; thus, we chose to avoid the risk of increasing the distress of participants with high levels of psychological suffering and/or with risky behaviors. The excluded participants were advised to seek professional help to deal with their symptoms/behaviors. Initially, there was an additional criterion for excluding participants in psychotherapy to avoid confounding; however, this criterion was withdrawn due to the high demand of participants in this situation. The effects of this confounding factor were considered in the statistical analyses.

**FIGURE 1:**
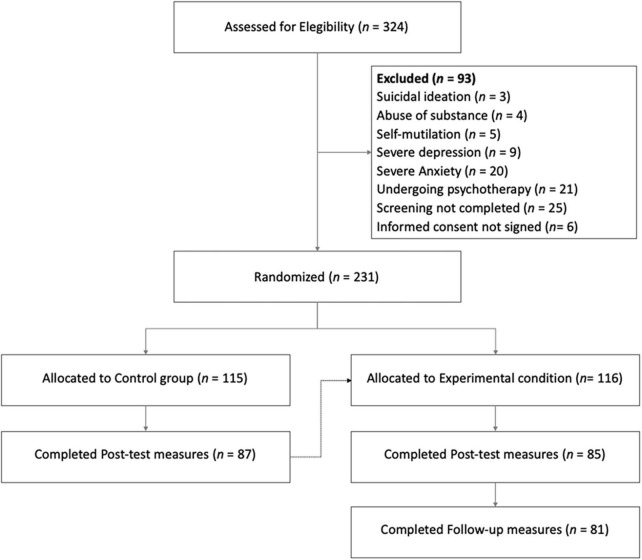
Participants’ flow from screening to follow-up.

Of the 324 participants who applied to the program, 62 were excluded at screening (see Figure 1 for details), 25 did not complete the screening, and 6 did not sign the informed consent form. The 231 participants who completed the screening and signed the informed consent were randomly assigned to the experimental group (*n* = 116) or to the control group (waiting-list) (*n* = 115). The drop-out rate was 26%, leading to 172 completers, 85 in the experimental group and 87 in the control group. The final sample size was considered adequate to detect an *f* effect size of 0.25, assuming α = 0.05, and statistical power of 0.9 and considering within and between interactions in a multivariate analysis of variance with two groups and two measurement points (Faul et al., 2007).

The participants’ mean age was 22.92 years (*SD* = 6.92). The majority of the sample was female (87%), Portuguese (89%), and single (94%), and attended the Psychology Master’s Degree program (66%). Although we had participants from 11 universities, most of them (91%) were from the University of Minho. The mean baseline symptomatology scores were mild anxiety (GAD-7; Spitzer et al., 2006), with *M* = 5.87 and *SD* = 3.81 and mild depression (PHQ-9; Kroenke and Spitzer, 2002), with *M* = 5.78 and *SD* = 3.81; 49% of the participants had psychotherapy in the past, and 10% were undergoing psychotherapy.

### Measures

***A sociodemographic questionnaire*** was developed within the scope of this study to obtain information on the sociodemographic characteristics of the sample, including relevant questions for screening (e.g., undergoing psychotherapy, presenting suicidal ideation and/or risk behavior).

***The Patient Health Questionnaire*** (PHQ-9) (Kroenke and Spitzer, 2002; adapted by Monteiro et al., 2013) is a 9-item self-report questionnaire assessing depression symptomatology. Participants rated symptoms experienced in the 2 weeks prior to the assessment on a 4-point Likert scale, from 0 (not at all) to 3 (nearly every day). Total scores range from 0 to 27 points, with a severity classification of minimal (0–4), mild (5–9), moderate (10–14), moderately severe (15–19), and severe (20–27). The Portuguese version showed an internal consistency of 0.86 (Monteiro et al., 2013).

***The Generalized Anxiety Disorder Scale*** (GAD-7) (Spitzer et al., 2006; adapted by Sousa et al., 2015) is a 7-item self-report questionnaire assessing symptoms of general anxiety. Participants rated symptoms experienced in the 2 weeks prior to the assessment on a 4-point Likert scale, from 0 (not at all) to 3 (nearly every day). Scores range from 0 to 21 points, with a severity classification of minimal (0–4), mild (5–9), moderate (10–14), and severe (15–21). The Portuguese version showed an internal consistency of 0.88 (Sousa et al., 2015).

***The Positive and Negative Affect Schedule*** (PANAS) (Watson et al., 1988; adapted by Galinha and Pais-Ribeiro, 2005) is a self-report measure assessing whether individuals experienced positive and negative emotions in the previous 2 weeks. Positive affect (PA) and negative affect (NA) scales each consist of 10 items. Responses to each item on a 5-point Likert scale ranged from 1 (very slightly or not at all) to 5 (extremely). Scale scores range from 10 to 50. Higher scores indicate higher levels of positive or negative affect. The Portuguese version showed internal consistency of 0.86 for the PA scale and 0.89 for the NA scale (Galinha and Pais-Ribeiro, 2005).

***The Revised Life Orientation Test*** (LOT-R) (Scheier et al., 1994; adapted by Laranjeira, 2008) is a self-report measure that assesses dispositional optimism. It consists of 10 items, of which only 6 are scored (items 1, 3, 4, 7, 9, and 10). The 4 items not scored are distractors and were not included in this study. Responses to each item are scored on a 5-point Likert scale, from 0 (strongly disagree) to 4 (strongly agree), with the total score ranging from 0 to 24. The Portuguese version showed an internal consistency of 0.71 (Laranjeira, 2008).

***The Satisfaction with Life Scale*** (SWLS) (Diener et al., 1985; adapted by Neto, 1993) is a 5-item self-report measure that assesses life satisfaction perception. Each item is rated on a 7-point Likert scale from 1 (strongly disagree) to 7 (strongly agree), with the total score ranging from 5 to 35. Higher scores indicate greater satisfaction with life. The Portuguese validation showed an internal consistency of 0.86 (Neto, 1993).

***The Ambivalence in Psychotherapy Questionnaire*** (APQ) (Oliveira et al., 2020) is a 9-item self-report measure that provides a global score of clients’ ambivalence levels toward change and two subscale scores: demoralization and wavering. Each item is rated on a 5-point Likert scale from 1 (totally disagree) to 5 (totally agree), with total scores ranging from 9 to 45. The demoralization dimension “emerges as the consequence of a perceived lack of skills to achieve change and the confusion of goals due to internal conflict” (Oliveira et al., 2020, p. 6) and consists of 5 items (e.g., “As much as I am sure of what I want to change, the next minute I feel lost”). The wavering dimension “refers to the oscillatory movements between two (or more) positions regarding a given object” (Oliveira et al., 2020, p. 6) and consists of 4 items (e.g., “Sometimes I think that everything will go well, and other times I think that everything will stay the same or get worse”). The APQ has excellent psychometric properties, with a Cronbach’s alpha value of 0.88 and test–retest reliability of r = 0.81. For this study, APQ instructions were adapted to the context of a writing intervention and the participants were asked to answer the questions reflecting on the problem they identified.

***The Ruminative Responses Scale*** (RRS-10) (Dinis et al., 2011) is a shorter version of the RRS (Treynor et al., 2003). This 10-item self-report measure assesses two rumination styles, brooding and reflection. Each item is rated on a 7-point Likert scale from 1 (almost never) to 7 (almost always). The reflection style consists of 5 neutral valence items and is interpreted as a way of engaging in contemplation, pondering, and coping to overcome the problem (e.g., “Go someplace alone to think about your feelings”) (Dinis et al., 2011). The brooding style indicates a thought process focused on the difficulties, obstacles, and inability to handle the situations and consists of five items (e.g., “Think ‘Why can’t I handle things better?”’) (Dinis et al., 2011). Participants were asked to respond to each question indicating what they usually do when they feel sad. The scale showed good internal consistency in the original version (α = 0.85) (Treynor et al., 2003) and in the Portuguese validation (α between 0.88 and 0.92, with 0.76 for the brooding style and 0.75 for the reflection style) (Dinis et al., 2011).

***The Outcome Questionnaire 10.2*** (OQ-10.2) (Lambert et al., 2005; Oliveira et al., 2019) is a 10-item self-report measure that monitors symptomatic change in the dimensions of psychological wellbeing (5 items) and psychological distress (5 items). Each item is rated on a 5-point Likert scale ranging from 0 (never) to 4 (almost always). Participants were asked to choose the alternative that better describes how they have been feeling during the last week, including today. Higher total scores indicate more symptomatic distress. The Portuguese validation showed an internal consistency of 0.80 (Oliveira et al., 2019).

***The Linguistic Inquiry and Word Count*** (LIWC) (Pennebaker et al., 2015) is a computerized text analysis software that quantifies the percentage of words falling into relevant categories for this study, namely: negative and positive emotion words (e.g., happy and sad), cognitive processing words (e.g., because and reason) and time orientation words (e.g., was, now, and going to). The LIWC was used to analyze the participants’ texts for a manipulation check of the writing instructions. The analysis sought to confirm whether the participants wrote emotionally and followed the instructions and to assess whether the different instructions resulted in different writing experiences.

The sociodemographic questionnaire was completed only at baseline. The PHQ-9 (Kroenke and Spitzer, 2002), GAD-7 (Spitzer et al., 2006), PANAS (Watson et al., 1988), LOT-R (Scheier et al., 1994), SWLS (Diener et al., 1985), APQ (Oliveira et al., 2020), and RRS-10 (Treynor et al., 2003) were used at baseline, posttest, and follow-up. The APQ (Oliveira et al., 2020), RRS-10 (Treynor et al., 2003), and OQ-10.2 (Lambert et al., 2005) were used after each writing task.

### Procedure

Write “n” Let Go was promoted through national and regional media, *via* social networks, through direct email from universities and their student associations, and at the University of Minho School of Psychology’ credits platform. The advertisements explained that the Write “n” Let Go program aimed to study the impact of internet-based writing tasks on university students’ psychological distress and wellbeing.

A web application was developed specifically for the intervention, enabling participants to complete enrollment and screening, sign online consent, fill in questionnaires, and perform the writing tasks online, using a computer or a mobile device.

The first step was registration, when the participants created a login and password required for future access to the application. Upon creating the account, the application prompted the screening questionnaires. After completing screening and signing the informed consent, the application randomly allocated participants either to the experimental group or to the control group, using the following procedure: check the number of participants in each group and allocate the participant to the group with fewer participants. If both groups had an equal number of participants, allocate the participant was allocated to a randomly chosen group.

The application then directed participants to the control panel, which was the default page displayed on subsequent logins. On this page, participants could read the program instructions, view the signed consent, withdraw from the program, send messages to the researchers, view the full schedule of activities and planned dates according to the group, and execute the activities. The “start task” button was enabled only on days when there was a planned activity. Upon clicking on the “start task,” the application workflow guided participants to carry out the activities planned for the day (the complete list of scheduled activities is provided as Supplementary Material).

Supporting videos were also used to guide participants throughout the program. The welcome video explained that the program consisted of four 20-min writing tasks on consecutive days related to a current unresolved problem causing distress that participants were required to identify next. The video reinforced that although the program was performed autonomously, participants should contact the researchers in case of intense or prolonged discomfort. For the control group, the welcome video included the information that the writing tasks would begin after a 2-week waiting period and highlighted the importance of that group in the study, as a way of motivating participants not to drop out. After watching the welcome video, participants of both groups filled in additional measures and identified a current problem. They also wrote a brief description of the problem, classifying its level of distress (low, medium, high, or very high) and category (personal, professional, relational, and/or familiar).

For the experimental group, the videos shown before the writing tasks described the main objective of each task, emphasizing its duration of 20 min and the importance of carefully reading the instructions. After reading the instructions, participants clicked on the “start writing” button and the application displayed a countdown clock with the remaining time for the task. The clock flashed when one minute remained. When the time expired the application closed the writing board and displayed the final text. At this point, no editing was possible, but participants could read their texts.

In the experimental group, measures were collected immediately after each writing task and 1 week (posttest) and 2 weeks (follow-up) after the last writing task. The control group participants were on a waiting list and responded to posttest measures 2 weeks after baseline. At this point, they started the combined writing tasks.

#### The Combined Writing Experimental Condition

The experimental condition consisted of 20 min writing tasks on 4 consecutive days related to the problem identified by the participant. Different instructions were presented each day. The main focus of each writing task was as follows: task 1—to express emotions, deepest thoughts and feelings about the problem identified; task 2—to organize thoughts, write about the problem’s origins, obstacles, consequences, maintenance factors; task 3—to identify resources, competencies, strengths and helpful resources to deal with the problem; and task 4—to imagine the life in the future with the problem solved or having stopped causing discomfort, and write about feelings and thoughts in that situation (the writing instructions are provided as Supplementary Material).

### Data Analysis

#### Preliminary Analysis

##### Controlling for Psychotherapy Effects

To control for the effects caused by accepting participants undergoing psychotherapy into the program, all analyses were replicated as follows: (1) paired sample *t*-tests, independent sample *t*-tests and chi-square tests were repeated excluding the participants undergoing psychotherapy; (2) for the MANOVAs, the undergoing psychotherapy condition was included as a between-subject factor.

##### Randomization Check

The experimental and control groups were compared for each measure collected at baseline using independent-sample *t*-tests. For the sociodemographic characteristics, in addition to the *t*-tests for age, chi-square tests were used to compare the categorical variables between groups. These analyses showed whether groups differed at baseline.

##### Dropout Profile

Randomization check analyses were repeated to assess dropout patterns in the experimental and control groups, comparing participants who completed the intervention with participants who dropped out.

##### Writing Topics

Descriptive statistics and chi-square tests compared the experimental and control groups in terms of writing topics, i.e., the category of the problem identified by participants and the perceived levels of distress caused by it.

##### Manipulation Check of Writing Instructions

The LIWC (Pennebaker et al., 2015) analyzed all participants’ texts to obtain the number of words per text and the percentage of words in the following categories: affect, negative emotions, positive emotions, cognitive processing, and time orientation (past, present, and future). ANOVA was conducted for each data extracted from LIWC to examine participants’ adherence to the instructions and the level of affect involved and to assess whether the different instructions resulted in different writing experiences.

#### Effects of the Program on Symptomatology and Wellbeing (Hypotheses 1 and 2)

The impact of the intervention on symptoms and related measures (anxiety, depression, ambivalence, and rumination—hypothesis 1) and on wellbeing (negative affect, positive affect, dispositional optimism, and satisfaction with life—hypothesis 2) were estimated using one-way repeated measures MANOVA (multivariate analysis of variance) comparing the baseline and posttest measures between the experimental and control groups. The same analysis was used to further evaluate the results at the subscale level, when applicable. For the variables with significant differences from baseline to posttest, paired-sample *t*-tests comparing the experimental group measures at posttest and follow-up were used to assess if the gains persisted at follow-up.

#### Effects of the Program Throughout the Writing Tasks (Hypothesis 3)

One-way repeated measures MANOVA was used to test if ambivalence, rumination, and psychological distress changed between the first and last writing tasks within the experimental group. *Post-hoc* pairwise comparisons using a Bonferroni correction for multiple comparisons assessed the impact of each pair of tasks individually.

#### Ambivalence Toward Change Mediation Analysis

An exploratory mediation analysis was performed with the experimental group. Statistical mediation requires four steps to be fulfilled (Baron and Kenny, 1986; Zhao et al., 2010): (1) the predictor (early symptomatology and wellbeing) is significantly related to outcome (later symptomatology and well-being); (2) the predictor is significantly related to the mediator (ambivalence, assessed between early and later measures); (3) the mediator is significantly related to outcome (later symptomatology and wellbeing); and (4) in the complete model, where the effect of the mediator is controlled for, the early symptoms and wellbeing effect on outcome is eliminated or significantly lessened. We applied the approach proposed by Zhao et al. (2010) with the Monte Carlo method (5,000 samples) to estimate standardized indirect effects with 95% confidence intervals.

Analyses were performed using IBM SPSS Statistics for Mac, Version 27.0, except for the mediation analysis, which used Stata 12 with the Medsem package (Mehmetoglu, 2018).

## Results

### Preliminary Analyses

#### Controlling for Psychotherapy Effects

Participants undergoing psychotherapy corresponded to 10% of the sample and were equally distributed between the experimental and control groups. The analyses performed to control for the effects of these participants on the overall program did not produce significant differences in reported results. Thus, the reported results include the entire sample.

#### Randomization Check

Table 1 presents the descriptive statistics of psychological measures collected at baseline, posttest, and follow-up as a function of group. Randomization check analyses did not produce any significant differences in baseline measures between the experimental and control groups (all *p*-values > 0.05), indicating successful randomization of participants. The sociodemographic characteristics at baseline did not differ between the groups (all *p*-values > 0.05).

**TABLE 1:** Descriptive statistics for psychological measures by group.

Psychological measure	Baseline			Posttest			Follow-up		
			
	*n*	*M*	*SD*	*n*	*M*	*SD*	*n*	*M*	*SD*
*Anxiety*									
Experimental	85	5.64	3.77	85	6.11	4.21	82	6.01	4.92
Control	87	6.10	3.85	87	6.20	4.47			
*Depression*									
Experimental	85	5.29	3.41	85	5.71	4.19	82	5.56	4.17
Control	87	6.26	4.14	87	6.37	4.68			
*Ambivalence*									
Experimental	85	30.16	7.11	85	25.31	7.90	81	25.20	7.96
Control	87	31.11	6.14	87	29.49	6.99			
*Rumination*									
Experimental	85	24.02	5.97	85	22.40	5.95	81	21.84	5.44
Control	87	22.89	4.91	87	22.82	5.25			
*Negative affect*									
Experimental	85	19.06	7.25	85	17.75	6.55	82	17.44	6.44
Control	87	18.99	7.35	87	17.79	6.60			
*Positive affect*									
Experimental	85	26.44	7.84	85	25.36	7.83	82	25.12	7.94
Control	87	27.62	7.74	87	25.98	7.35			
*Dispositional optimism*									
Experimental	85	14.01	4.89	85	14.04	5.19	82	14.20	5.07
Control	87	14.75	4.32	87	14.93	4.58			
*Satisfaction with life*									
Experimental	85	17.18	3.48	85	17.61	3.73	81	17.60	3.66
Control	87	17.48	3.35	87	17.84	2.94			

*Empty cells are measures not collected at follow-up for the control group.*

#### Dropout Profile

Participants from the experimental group who dropped out presented on average higher depression (*p* = 0.02) and lower life satisfaction (*p* = 0.016) baseline scores than participants who completed the intervention. No other significant differences were found in the experimental group for baseline measures or sociodemographic characteristics (all *p*-values > 0.05). No patterns of dropout were found in the control group’s participants. Most of the dropouts (73%) occurred after the initial activity of identifying the problem.

#### Writing Topics

Table 2 presents the participants’ categorization of their identified problems. The chi-square test results confirmed no significant association between group and problem category. In both groups, participants perceived their difficulties mostly as personal, although 43% selected more than one category in this answer. Some examples of the topics identified were grief, physical disability, obesity, childhood sexual abuse, sexuality, job loss, loneliness, depression, stress with the university, test anxiety, choice of career, end of a relationship, low self-esteem, problems with parents, serious family illness, COVID-19 pandemic confinement, among others.

**TABLE 2:** Problem category by group.

Problem category	Experimental %	Control %
Personal	41%	39%
Professional	20%	20%
Relational	18%	25%
Familiar	19%	17%
Other	3%	–

Table 3 presents the participants’ perceived level of distress caused by the problem as a function of group. The chi-square test results confirmed a significant relationship between group and the perceived level of distress caused by the problem [*X*^2^(1, *N* = 210) = 5.90, *p* = 0.015]. Participants in the experimental group were more likely to report a problem of high/very high distress levels than those in the control group. Moreover, less than 10% of participants considered their problem to have a low distress level, indicating that most participants chose a relevant and distressing problem as a writing topic.

**TABLE 3:** Problem distress level by group.

Problem distress level	Experimental %	Control %
Low	4.7	9.2
Moderate	25.9	35.6
High	38.8	41.4
Very High	30.6	13.8

#### Manipulation Check of Writing Instructions

Table 4 displays the descriptive statistics of the participants’ texts from the experimental group. An ANOVA confirmed that the different writing instructions resulted in systematic differences in the writing experience, suggesting that participants adhered to the instructions. There were, on average, 336 words per text (*SD* = 118), and the first two writing tasks had a significantly higher number of words than the last two. The percentage of affect words did not differ across tasks; however, an average of 6.9% is comparable to other expressive writing experiences reported in the LIWC manual (4.8%) (Pennebaker et al., 2015), indicating that participants wrote emotionally.

**TABLE 4:** Words in participants’ texts as a function of writing task.

	Writing task 1	writing task 2	Writing task 3	Writing task 4
Total words	413 (178)_a_	354 (190)	313 (184)_b_	264 (179)_b_
% of affect	6.5 (1.9)_a_	6.5 (1.9)_a_	6.9 (2.1)_a_	7.9 (3.0)_a_
% of positive emotion	2.7 (1.1)_a_	3.0 (1.1)_a_	4.0 (1.7)_b_	5.0 (2.4)_*c*_
% of negative emotion	3.5 (1.6)_a_	3.4 (1.4)_a_	2.7 (1.3)_b_	2.9 (1.6)_b_
% of cognitive processing	21.6 (3.3)_a_	22.0 (3.4)_a_	21.6 (4.4)_a_	21.3 (4.8)_a_
% of focus past	3.0 (2.4)_a_	2.5 (1.8)	1.8 (1.8)_b_	1.9 (1.7)_b_
% of focus present	9.3 (2.8)_a_	9.3 (2.4)_a_	9.7 (2.8)_a_	7.3 (2.9)_b_
% of focus future	1.0 (0.9)_a_	1.1 (1.0)_a_	0.9 (0.8)_a_	1.6 (1.6)_b_

*Values for the experimental group (n = 85) are reported as M (SD). Means in the same rows with different subscripts differ at the p = 0.01 level by Bonferroni pairwise comparisons. Means with the same or no subscript do not significantly differ.*

As predicted, in the first two writing tasks the average negative emotion words were significantly higher than in the last two tasks. Concurrently, the last two tasks had a significantly higher average of positive emotion words than the first two tasks. Although the cognitive processing words did not differ across the tasks, an average of 21.6% is higher than expected in the context of expressive writing experiences (12.5%) (Pennebaker et al., 2015), indicating that the instructions succeeded in promoting cognitive activity. In terms of time orientation, as expected, the first two tasks were significantly more focused on the past than the last two tasks, the first three tasks were significantly more focused on the present than the fourth task, and the fourth task was significantly more focused on the future, than the first three tasks.

### Effects of the Program on Symptomatology (Hypothesis 1)

The hypothesis that the writing program would reduce depression, anxiety, rumination, and ambivalence in the participants of the experimental group when compared with the control group was partially confirmed. The MANOVA indicated a significant effect on the group x time interaction [Pillai’s Trace = 0.129, *F* (4, 167) = 6.20, *p* < 0.001, η*_p_*^2^ = 0.13]. From a univariate perspective, both ambivalence and rumination were significantly lower in the experimental group than in the control group, but no differences were found in depression and anxiety (see Table 5 for detailed MANOVA univariate results). Assessing the subscales level with the MANOVA, rumination univariate results showed that only the brooding subscale reduced significantly [*F*(1, 171) = 6.84, *p* = 0.010, η*_p_*^2^ = 0.04], whereas for ambivalence, both the wavering [*F*(1, 170) = 7.99, *p* = 0.005, η*_p_*^2^ = 0.05] and the demoralization [*F*(1, 170) = 13.66, *p* < 0.001, η*_p_*^2^ = 0.07] subscales reduced significantly. MANOVA assumptions were met.

**TABLE 5:** One-way repeated measures MANOVA between groups for symptoms and related measures.

Psychological measure	*M* Experimental		*M* Control		*F*(1, 170)	*p*	η_p_^2^
		
	Baseline	Posttest	Baseline	Posttest			
Anxiety	5.64	6.11	6.10	6.20	0.52	0.471	0.003
Depression	5.29	5.71	6.26	6.37	0.36	0.548	0.002
Ambivalence	30.16	25.31	31.11	29.49	16.97	<0.001	0.091
Rumination	24.02	22.40	22.89	22.82	6.47	0.012	0.037

*All p-values in this table are two-tailed.*

Paired-sample *t*-tests within the experimental group resulted in no significant differences between post-test and follow-up on ambivalence (*t* (80) = 0.289, *p* = 0.774) and rumination [*t*(80) = 0.71, *p* = 0.480], confirming that the gains obtained persisted at follow-up.

### Effects of the Program on Wellbeing (Hypothesis 2)

The writing program did not produce an effect on wellbeing measures. The MANOVA confirmed a non-significant effect on the group x time interaction [Pillai’s Trace = 0.005, *F*(4, 168) = 0.206, *p* = 0.935, η*_p_*^2^ = 0.05]. From a univariate perspective, no differences between groups were found in negative affect, positive affect, dispositional optimism, and satisfaction with life (see Table 6 for detailed MANOVA univariate results). MANOVA assumptions were met.

**TABLE 6:** One-way repeated measures MANOVA between groups for wellbeing measures.

Psychological measure	*M* Experimental		*M* Control		*F*(1, 171)	*p*	η_p_^2^
		
	Baseline	Posttest	Baseline	Posttest			
Negative affect	19.06	17.75	19.07	18.13	0.17	0.684	0.001
Positive affect	26.44	25.36	27.57	25.90	0.50	0.479	0.003
Dispositional optimism	14.01	14.04	14.69	14.83	0.08	0.775	0.000
Satisfaction with life	17.18	17.61	17.35	17.72	0.06	0.814	0.000

*All p-values in this table are two-tailed.*

### Effects of the Program Throughout Writing Tasks (Hypothesis 3)

A one-way repeated measures MANOVA indicated that ambivalence, rumination, and psychological distress decreased throughout the writing tasks, supporting the 3rd hypothesis. Significant differences were found in mean vectors among the 1st, 2nd, 3rd, and 4th tasks across the 3 dependent variables, Pillai’s Trace = 0.445, *F*(9, 77) = 9.70, *p* < 0.001, η*_p_*^2^ = 0.45. From a univariate perspective, significant reductions between the 1st and the 4th tasks were found in ambivalence, *F*(3, 255) = 26.26, *p* < 0.001, η*_p_*^2^ = 0.24, in psychological distress, *F*(3, 255) = 14.45, *p* < 0.001, η*_p_*^2^ = 0.15, and in rumination *F*(3, 255) = 4.84, *p* = 0.003, η*_p_*^2^ = 0.05. In *post-hoc* pairwise comparison analyses using a Bonferroni correction (see Table 7 for detailed results), ambivalence was significantly lower in task 2 than in task 1 (*p* = 0.001) and in task 3 than in task 2 (*p* = 0.024). Psychological distress was significantly lower in task 3 than in task 2 (*p* = 0.001). Rumination, in turn, did not show significant reductions in any pair of tasks specifically. MANOVA assumptions were met.

**TABLE 7:** Pairwise comparisons for measures collected after the writing tasks.

Psychological measure	(I) Task	(J) Task	Difference in means (I–J)	*f*	*p* ^a^	95% CI for difference in means^a^	
						
						LL	UL
Psychological distress	1	2	0.372	0.267	1.000	–0.348	1.093
		3	1.419*	0.309	<0.001	0.583	2.254
		4	1.674*	0.353	<0.001	0.722	2.627
	2	3	1.047*	0.260	0.001	0.343	1.750
		4	1.302*	0.313	<0.001	0.456	2.149
	3	4	0.256	0.289	1.000	–0.525	1.036
Ambivalence	1	2	1.442*	0.368	0.001	0.448	2.436
		3	2.407*	0.370	<0.001	1.408	3.406
		4	3.163*	0.453	<0.001	1.938	4.387
	2	3	0.965*	0.326	0.024	0.086	1.845
		4	1.721*	0.416	<0.001	0.597	2.845
	3	4	0.756	0.307	0.095	–0.073	1.584
Rumination	1	2	0.535	0.285	0.386	–0.236	1.306
		3	0.953*	0.337	0.035	0.044	1.863
		4	1.058*	0.347	0.018	0.122	1.995
	2	3	0.419	0.244	0.543	–0.242	1.079
		4	0.523	0.324	0.663	–0.353	1.400
	3	4	0.105	0.305	1.000	–0.721	0.930

*Based on estimated marginal means.*

*CI, confidence interval; LL, lower limit; UL, upper limit.*

*The difference in means is significant at the 0.05.

*^a^Adjustment for multiple comparisons: Bonferroni.*

### Ambivalence Toward Change Mediation Analysis

For the first set of analyses, the independent variable was task 1 OQ-10.2, the mediator was task 2 APQ (when the first significant decrease in ambivalence occurred), and the dependent variable was task 4 OQ-10.2. Pathway coefficients suggested significant associations between task 1 OQ-10.2 and task 2 APQ (β = 0.791, *p* < 0.001), between task 2 APQ and task 4 OQ-10.2 (β = 0.107, *p* = 0.010), and between task 1 OQ-10.2 and task 4 OQ-10.2 (β = 0.772, *p* < 0.001). Consistent with the fourth assumption condition of mediation analysis, the variance in task 4 OQ-10.2 scores explained by task 1 OQ-10.2 was reduced after controlling for task 2 APQ with ambivalence explaining approximately 10% of the effect of task 1 OQ-10.2 on task 4 OQ-10.2 (see Table 8 for details).

**TABLE 8:** Standardized indirect effects with 95% confidence intervals.

Model pathways	Indirect effect	Standard error	95% CI			
			
			Lower	Upper	*z*	*p*
Task 1 PD → Task 2 Ambivalence → Task 4 PD	0.085	0.034	0.018	0.151	2.486	0.013
Task 1 Rumination → Task 2 Ambivalence → Task 4 Rumination	0.116	0.036	0.045	0.188	3.201	0.001
Baseline Rumination → Task 2 Ambivalence → Posttest Rumination	0.089	0.035	0.020	0.157	2.547	0.011

*PD, Psychological distress.*

In a second set of analyses, the mediation role of ambivalence on the effects of early rumination (baseline and task 1) on later rumination (task 4 and posttest) was tested. First, pathway coefficients suggested significant associations between baseline RRS-10 and task 2 APQ (β = 0.727, *p* < 0.001), between task 2 APQ and posttest RRS-10 (β = 0.122, *p* = 0.007), and between baseline RRS-10 and posttest RRS-10 (β = 0.676, *p* < 0.001). A similar pattern was found on the pathways between Task 1 RRS-10 and Task 2 APQ (β = 0.889, *p* < 0.001), between task 2 APQ and task 4 RRS-10 (β = 0.131, *p* = 0.001), and between task 1 RRS-10 and task 4 RRS-10 (β = 0.764, *p* < 0.001). In both models, the variance in later rumination scores explained by early rumination was reduced after controlling for task 2 APQ, with ambivalence explaining approximately 12% of the effect of baseline RRS-10 on posttest RRS-10, and 13% of the effect of task 1 RRS-10 on task 4 RRS-10 (see Table 8 for details).

## Discussion

As referred previously, the efficacy of WBIs has received mixed support. A fundamental aspect that has been investigated is the experimental manipulation of the writing instructions. One of the approaches in this regard is the use of different instructions, guiding participants to change their focus, even when writing about the same topic.

Write “n” Let Go used this approach and combined different instructions, centering the two initial tasks on the participants’ identified problem and the two subsequent tasks on its resolution. These problems encompassed intrapsychological (e.g., anxiety about exams and demotivation), interpersonal (e.g., conflicts with parents or peers) and contextual elements, such as the first COVID-19 confinement in Portugal at the time in which the program was implemented.

The results partially confirmed the efficacy of the intervention. Between-group comparisons indicated that while rumination and ambivalence significantly decreased from baseline to post-test in the experimental group when compared with the control group, no changes were detected in measures of symptoms and wellbeing. Gains in ambivalence and rumination were maintained at follow-up.

One possible interpretation for the absence of changes in depression, anxiety, satisfaction with life, affect, and optimism is that writing about a current problem may not necessarily promote wellbeing and symptomatology decrease in the short term, especially as most participants perceived their problems as highly distressing. However, the significant decreases in rumination and ambivalence suggest that the intervention managed to target aspects directly associated with the participants’ problem, soothing the conflicting perspectives (i.e., ambivalence) and the tendency to ruminate (and especially to brood). This notion is supported by the significant decreases in ambivalence, rumination, and psychological distress detected across the writing tasks. Psychological distress was measured with the OQ-10.2 (Lambert et al., 2005), which assesses a general degree of wellbeing and distress, indicating that some improvements were achieved through writing, although no effects were detected on measures of symptoms and wellbeing between groups. In addition, changes in a relevant cognitive factor, such as rumination, in the long run have the potential to prevent an increase in symptoms, especially in a sample with mild baseline symptoms. Although the effects detected on ambivalence and rumination are promising, they should be interpreted with caution since our sample was composed mostly of psychology students who may be more conscious about their problems and highly motivated to reflect on them. This possible bias of our sample can also explain the higher use of cognitive processing words, when compared with other studies (Pennebaker et al., 2015).

The measures collected after each task also enabled an exploratory analysis of mediation to test whether ambivalence could explain the effects of the intervention. The analysis showed that ambivalence partially mediated the changes in distress and rumination. These results must also be interpreted with caution, considering that the same measures were used as predictors (e.g., baseline rumination) and response variables (e.g., posttest rumination) and that the analysis included the experimental group only. Nonetheless, the results contribute to the notion that ambivalence toward change may also be an important factor associated with WBIs efficacy, at least in the context of combined instructions focused on a current problem.

Write “n” Let Go started by prompting participants to identify a current unresolved problem that may be charged with ambivalence and thus accompanied by the feeling of being stuck. The initial writing tasks may have lowered this feeling, allowing participants not only to express the consequences of their problems but also to ponder about what sustains it and the consequences of its resolution in a secure environment without any pressure to “make up their minds.” Complementarily, the last writing tasks changed focus, allowing participants to reflect on resources and strengths to handle the problem and simulate successful outcomes. This integrative perspective may have created adequate conditions for participants to feel more resourceful and resolve ambivalence. Motivated to promote changes, they could let go of ruminating about their difficulties and feel less stressed. In short, our hypothesis is that the decrease in ambivalence towards change could have interacted with other processes at play (emotional ventilation, exposure, self-regulation, and cognitive processing) to favor these improvements.

Although we speculate that these outcomes may result from our choice of design, testing a more thorough design is required to draw concrete conclusions and assess the differential efficacy between the use of combined instructions and individual instructions. Nonetheless, our findings are consistent with the views of writing as a way to change perspectives, stand back, re-evaluate life situations, and develop a better understanding of the problem (Pennebaker and Chung, 2007), resulting in greater coherence of thoughts and emotions (Welch et al., 2020) and increased self-regulation and sense of control over life’s challenges, thus with the potential to address internal conflicts and reduce associated symptoms such as rumination and distress.

### Limitations

Our study had several important limitations. As mentioned, we did not compare the combined writing with the EW and PW instructions individually. The risk of an underpowered study, given the sample size required to run a more robust design (i.e., with four groups) led us to run a preliminary assessment of the program’s effect first, impeding more concrete conclusions. Although some studies have proven the incremental efficacy of combined writing, this aspect should be systematically tested in future studies.

Another limitation was the option for a passive control group (waiting-list) instead of having participants write about a neutral topic. This would entail a more straightforward comparison between the groups, given that both would perform writing tasks and have measures collected after the tasks. The reasons for this option were mainly ethical. We wanted to give all participants the possibility of doing the writing tasks, which the control group could do after the waiting period. At the same time, we considered that having participants write about a neutral topic after the identification of a problem at baseline could create a source of distress. Future studies should attempt to find ways to use active control groups, with some sort of writing task involved.

In terms of sample selection, several aspects restrain the generalization of our findings and require improvement. First, a more heterogeneous sample with students from different universities and areas of knowledge would provide more comprehensive evidence on the efficacy of this program. Our sample was mainly composed of women and psychology master’s students, who may be prone to reflect upon their emotions and thoughts, and eventually more receptive to our writing instructions. In addition, 77% of the completers gained college credits, thus involving extrinsic motivation for participating. Second, the effects of the intervention in participants with severe anxiety and severe depression are unknown. Since the program was being tested for the first time, we decided to exclude participants with severe symptomatology due to ethical concerns so as not to risk aggravating their condition. Last, although we have accomplished successful randomization between groups in terms of sociodemographic characteristics and baseline measures, the participants from the experimental group perceived their problems as more distressing than the control group participants, which may have influenced the results.

Considering the questionnaires used and the moments of assessment, several challenges were posed. The choice of short questionnaires for depression (PHQ-9; Kroenke and Spitzer, 2002) and anxiety (GAD-7; Spitzer et al., 2006), instead of longer ones, and more often used in research on WBIs, may have limited our capacity to detect small symptom changes. The absence of follow-up assessment in the control group also impeded the comparison of the groups at the three assessment points (baseline, posttest and follow-up). As referred, control participants were to perform the intervention after the waiting period, and we considered that a 3-week waiting period would be too long and discouraging.

Although using an online format allowed us to reach university students across the country, it impeded control of the conditions under which the writing tasks were performed. Nonetheless, the manipulation check provided positive indications of adherence to instructions, suggesting the feasibility of delivering WBIs over the internet. The patterns of dropout, however, may indicate that more distressed participants may benefit from closer support from the researchers or from a more attentive program briefing during the welcome video, especially considering that most dropouts occurred after the participants identified their problems.

### Implications

Despite the limitations, our study presents preliminary evidence of the combined writing efficacy and emphasizes the importance of studying ambivalence toward change in WBIs. Balancing EW and PW instructions may be a way to foster interactions between disparate processes, which may be more aligned with the participants’ cognitive style and/or be more adequate for a given life difficulty. The early decrease in ambivalence may signal that ambivalence resolution could facilitate other processes, such as cognitive processing or self-regulation, by allowing participants to clarify their goals and foster a better integration of life difficulties into their meaning schemas. In sum, our results emphasize the relevance of further investigation of motivational factors in WBIs, in addition to supporting the argument that multiple interacting processes are likely driving its efficacy (Sloan and Marx, 2004; Smyth and Pennebaker, 2008).

Future studies could test combined writing with more robust designs and with different populations to allow for concrete conclusions. Additionally, ambivalence toward change could be systematically investigated to confirm its potential mediating role in writing-based interventions that target a current unresolved problem for which an adaptive response is needed to avoid suffering and negative consequences.

The confirmation of our findings can be an aspect in favor of the widespread use of this methodology, given the low cost involved. As ambivalence toward change is a key variable to foster change in psychotherapy (Oliveira et al., 2021a), a writing protocol could become an entry solution for patients on the psychotherapy waiting list, following a stepped care approach (Davison, 2000). Moreover, offering online WBIs has the potential to reach many students. There is a high prevalence of university students diagnosed with mental disorders, and the COVID-19 pandemic exacerbated this situation. Mental disorders are associated with substantial delays in treatment and tend to have an early adulthood onset, coinciding with the challenging period at university (Duffy et al., 2019). Thus, WBIs have the potential to be powerful tools for the early detection of students at risk of developing emotional disorders. Furthermore, given the anonymous context and accessible nature, WBIs could help raise awareness of the importance of mental health among students and become an alternative for those who are averse or do not have access to traditional psychological treatments.

## Data Availability Statement

The raw data supporting the conclusions of this article will be made available by the authors upon request.

## Ethics Statement

The studies involving human participants were reviewed and approved by the Research Ethics Committee in Social and Human Sciences of the University of Minho. The patients/participants provided their written informed consent to participate in this study.

## Author Contributions

JB: conceptualization, manuscript preparation, writing (sections “Introduction” and “Discussion”), and reviewing and editing of text. JM: conceptualization, data curation, writing (sections “Introduction” and “Results”), and reviewing and editing of text. MG: data curation and writing of section “Method.” JO: data analysis, writing of “Results” and “Introduction” sections on “Ambivalence Toward Change.” MMG: conceptualization, and reviewing and editing of text. All authors contributed to the article and approved the submitted version.

## Conflict of Interest

The authors declare that the research was conducted in the absence of any commercial or financial relationships that could be construed as a potential conflict of interest.

## Publisher’s Note

All claims expressed in this article are solely those of the authors and do not necessarily represent those of their affiliated organizations, or those of the publisher, the editors and the reviewers. Any product that may be evaluated in this article, or claim that may be made by its manufacturer, is not guaranteed or endorsed by the publisher.
