# Supplementary material for: Write and Let Go: An Online Writing Program for University Students
Source: Front Psychol. 2022 Jul 7;13:874600. doi: 10.3389/fpsyg.2022.874600 (PMC9301038; doi:10.3389/fpsyg.2022.874600)
Supplement: Supplementary file 1 [file Data_Sheet_1.docx]

**Instructions for the writing tasks (translated from Portuguese)**

**Task 1 - Express your emotions**

*For the next 20 minutes, we would ask you to please write about your innermost thoughts and feelings regarding the problem you identified previously.*

*You can associate your writing topic with how you behave when faced with the problem or even with your relationships with others, including parents, romantic partners, friends, or family; you can write about how the problem you identified has affected you in the past, affects you now, or will affect you in the future, as well as how it affects who you have been, who you would like to be, or who you are today.*

*The important thing is that you let go and explore your deepest thoughts and emotions. Don't worry about sentence structure, grammatical or spelling errors, it's not important. The only rule is that once you start writing, you must keep doing it until the time runs out.*

**Task 2 - Organize your thoughts**

*For the next 20 minutes, we would ask you to please continue writing about the same problem that you identified.*

*You can associate your writing topic with the description of the problem, including the factors associated with its origin, as well as the obstacles that prevent its resolution and the factors that contribute to its maintenance. You can write about the likely consequences if the problem is resolved. You can also write about the role of relationships with others in this problem, including parents, romantic partners, friends, or family.*

*As you write, we would like you to let go and explore your deepest thoughts and emotions. Don't worry about sentence structure, grammatical or spelling mistakes, it's not important. The only rule is that once you start writing, you must keep doing it until the time runs out.*

**Task 3 - Recognize your strengths**

*During the next 20 minutes, we would ask you to please write about your innermost thoughts and feelings regarding your skills, strengths, and resources.*

*You can reflect on how these skills can be useful to you in solving your current problem. You can also link the skills to problems that are similar to what you are experiencing today. If it is difficult to identify resources, you can write about how you have dealt with other difficulties in the past and how your strengths have been useful. You can associate your writing topic with your relationships with others, including parents, romantic partners, friends, or family.*

*As you write, we would like you to let go and explore your deepest thoughts and emotions. Don't worry about sentence structure, grammatical or spelling errors, it's not important. The only rule is that once you start writing, you must keep doing it until the time runs out.*

**Task 4 - Imagine the problem solved**

*During the next 20 minutes, we would ask you to please think about your life in the near future, in which the problem you identified was resolved or that no longer caused such great discomfort and to write about your innermost thoughts and feelings in this case.*

*Imagine that everything went as smoothly as possible, and you were able to better deal with the problem or resolve it properly. Try to describe in detail and intensely what that situation would be like and the associated emotions and thoughts, how it would be different to have this problem solved or how you tried to achieve it. You can associate your writing topic with your relationships with others, including parents, romantic partners, friends, or family.*

*As you write, we would like you to let go and explore your deepest thoughts and emotions. Don't worry about sentence structure, grammatical or spelling errors, it's not important. The only rule is that once you start writing, you must keep doing it until the time runs out.*
