# Supplementary material for: Write and Let Go: An Online Writing Program for University Students
Source: Front Psychol. 2022 Jul 7;13:874600. doi: 10.3389/fpsyg.2022.874600 (PMC9301038; doi:10.3389/fpsyg.2022.874600)
Supplement: Supplementary file 2 [file Table_1.docx]

Scheduled Activities by Group

| Group | Planned date | Activities workflow (in the order presented to participants) |
| --- | --- | --- |
| Experimental | Day 0 | - watch the welcome video - collect baseline measures (LOT-R, PANAS, SWLS, RRS-10) - think about a problem - write a brief description (1 line) of the problem - register the problem’s distress level - register the problem’s category - collect baseline measures (APQ) |
|  | Day 1 | - watch the 1^st^ writing task video - perform 20-min writing task “Express emotions” - collect after session measures (OQ-10.2, RRS-10, APQ) |
|  | Day 2 | - watch the 2^nd^ writing task video - perform 20-min writing task “Organize thoughts” - collect after session measures (OQ-10.2, RRS-10, APQ) |
|  | Day 3 | - watch the 3^rd^ writing task video - perform 20-min writing task “Recognize strengths” - collect after session measures (OQ-10.2, RRS-10, APQ) |
|  | Day 4 | - watch the 4^th^ writing task video - perform 20-min writing task “Imagine the problem solved” - collect after session measures (OQ-10.2, RRS-10, APQ) |
|  | Day 11 | - collect posttest measures (PHQ-9, GAD-7, LOT-R, SWLS, PANAS, RRS-10, APQ) |
|  | Day 18 | - collect follow-up measures (PHQ-9, GAD-7, LOT-R, PANAS, SWLS, RRS-10, APQ) |
| Control | Day 0 | - watch the welcome video - collect baseline measures (PHQ-9, GAD-7, LOT-R, PANAS, SWLS, RRS-10, APQ) - think about a problem - write a brief description (1 line) of the problem - register the problem’s distress level - register the problem’s category - collect baseline measures (ambivalence) |
|  | Day 14 | - collect follow-up measures (PHQ-9, GAD-7, LOT-R, PANAS SWLS, RRS-10, APQ) |
